# Institutions and institutional changes: aquatic food production in Central Luzon, Philippines

**DOI:** 10.1007/s10113-021-01853-4

**Published:** 2021-12-02

**Authors:** Aisa O. Manlosa, Anna-Katharina Hornidge, Achim Schlüter

**Affiliations:** 1 Social Sciences Department, Leibniz Centre for Tropical Marine Research (ZMT), Fahrenheitstraße 8, 28359 Bremen, Germany; 2 German Development Institute / Deutsches Institut für Entwicklungspolitik (DIE), Tulpenfeld 6, 53113 Bonn, Germany; 3 Institute of Political Sciences and Sociology, University of Bonn, Regina-Pacis-Weg 3, 53113 Bonn, Germany; 4 Department of Business and Economics, Jacobs University, Campus Ring 1, 28759 Bremen, Germany

**Keywords:** Blue economy, Blue food, Mariculture, Social change, Sustainability, Transformation

## Abstract

Aquaculture is the most rapidly growing food production sector globally. In certain coastal social-ecological systems, this has resulted in significant changes and sustainability challenges. In particular, coastal environments which used to support only capture fisheries are becoming sites for brackish water aquaculture production; this impacts the sustainability of aquatic food production. Sustainability challenges associated with aquaculture expansion and intensification necessitate a contextually rooted understanding of institutions and institutional changes which can be used as an informed basis for leveraging institutions to achieve desirable sustainability outcomes in the aquatic food sector. This research used a qualitative empirical case study involving in-depth interviews, participant observation, and analysis of institutional documents in the region of Central Luzon, Philippines. It applied the inter-institutional systems concept which considers multiple institutions with distinct but linked purposes and functions in the societal spheres of state, market, and civil society. The study found that aquaculture emerged as an important livelihood because of rice farmers’ need to adapt to saltwater intrusion into what were formerly rice farms. It grew into an industry due to developments in the availability and accessibility of inputs such as fingerlings and feeds. This process was also driven by the high demand and high profitability of fish farming at the time. Regulatory institutions have not adequately adapted to protect the environment. Market institutions adapted but the changes mostly benefited consignacions (middlemen) and large-scale players. However, organised groups of collaborating smallholder fishers and fish farmers are helping to address the disadvantages they face.

**Supplementary Information:**

The online version contains supplementary material available at 10.1007/s10113-021-01853-4.

## Introduction

Globally, the total production of aquatic food is increasingly coming from aquaculture. Aquaculture now provides more than half of fish proteins for human consumption (FAO 2020). In certain parts of the world, coastal social-ecological systems understood as complex coupled human–environment systems (Berkes et al. 1998), are undergoing a shift from one where most production comes from capture fisheries to one where aquaculture dominates production. Such shifts raise sustainability concerns related to pressure and conflict around coastal resources (Bavinck et al. 2018), water pollution (Duarte et al. 2009), biodiversity effects (Diana 2009), benefit distribution (Salayo et al. 2012), and nutritional implications (Belton and Thilsted 2014), among others.

Institutions^1^ which encompass formal and informal rules play a key role in the governance of aquatic food production,^2^ particularly in addressing sustainability challenges in coastal spaces (Jentoft 2004; Morgan et al. 2017; Campbell et al. 2021). In the past decade, institutions have increasingly figured in discourses concerning transformations to sustainability (e.g. Galaz et al. 2012; Westley et al. 2013). This is not only because institutions influence windows of opportunities for sustainability solutions but also because they are key to scaling up new practices (Nyborg et al. 2016) and to establishing new social-ecological system regimes. From a systems perspective, institutions have been considered leverage points for transformative systemic change (Meadows 1999; Abson et al. 2016). The levers for transformation identified in the recent Global Sustainable Development Report which include governance, individual action, and collective action (Messerli et al. 2019) are all shaped by institutions in one way or another.

In the aquaculture sector, studies on institutions and institutional change predominantly focus on either designed rules typically implemented at the fish-farm level (e.g. certification, finance, and insurance) (Kalfagianni and Pattberg 2013; Bush et al. 2019a) or on formal state regulations. While each is important, new institutional arrangements designed to address sustainability problems do not operate in a vacuum and are not free from the influence of political dynamics and power relations (sensu Chang 2007; Cleaver and de Koning 2015). Such new arrangements enter an existing system of institutions where configurations of formal and informal rules already exist, interact, and change at different paces due to different drivers, in different ways, within matrices of social relations (Shand 2015). This relatively stable but also dynamic system of interacting rules (Mahoney and Thelen 2010) influences sustainability outcomes in coastal social-ecological systems and similar settings.

In view of an incomplete understanding of the way that multiple institutions contribute to addressing or reproducing sustainability challenges in the aquatic food sector, place-based institutions research can inform governance particularly in coastal social-ecological systems that are increasingly dominated by aquaculture production. This is urgently needed as aquaculture has grown exponentially over the last decades and has become the dominant mode of aquatic food production in Asia, with comparatively less attention from sustainability and institutional research. A similar trend is unfolding in Africa (FAO 2020) where ecological, social, and economic sustainability shortcomings may yet be avoided.

We used a case study approach focusing on the Central Luzon region in the Philippines, an important aquatic food-producing country (FAO 2020). Central Luzon is among the country’s most economically valuable aquaculture-producing areas (Philippine Statistics Authority 2020). The aim of this study is to build an empirical understanding of institutions and institutional change processes in a context where there is traditionally high dependence on capture fisheries, and increased importance of aquaculture. The scope covers the societal spheres of state, market, and civil society (Jentoft 2004; Kooiman 2008). In each of these spheres, we address the following objectives: (1) identify and describe the institutions that underpin aquatic food production; (2) explain whether and how institutions changed as aquaculture grew and became an increasingly important aquatic food sector; and (3) determine key factors that were influential to the institutional changes. The study’s contribution to sustainability discourse is unpacked in the discussion section. The conclusion focuses on concrete implications for governance in the case study context.

## Conceptual framework

Understanding how institutions change and how they lead to outcomes in social-ecological systems is important for facilitating systemic change towards sustainability. Social scientists defined the term institutions in various ways depending on what they consider to be the most important aspect of human interactions (Jentoft 2004; Hodgson 2006). We adopted the broad definition of institutions as a set of rules that structure social interactions (Hodgson 2006). Institutions exist in different forms (Jentoft 2004; Hodgson 2006). On one hand, they may be formal and codified at different scales such as national laws and local regulations and enforced through monitoring systems, penalties, or incentives. Marine Protected Areas and their associated rules (MPAs) are examples of formal institutions in coastal social-ecological systems (Gruby et al. 2021). On the other hand, institutions may be informal such as social norms and conventions (Nonaka 1994). Informal institutions are typically tacit and are maintained and reproduced by being imbibed into ways of thinking and repeated social practice at the individual and community levels (Manlosa 2019). Examples include gender norms in aquaculture and coastal settings (Weeratunge et al. 2010; Kruijssen et al. 2018) and customary resource management systems (Galappaththi and Nayak 2017). In some cases, informal institutions are later formalised. This happens when laws are created to provide legal recognition and protection of indigenous rule systems in hybrid management systems (Cinner and Aswani 2007; Campbell et al. 2012).

Here, we conceptualise the aquatic food sector as one that is embedded in an inter-institutional system (Thornton et al. 2015) consisting of formal and informal rules with distinct but linked purposes and functions. We apply this concept in our study through the inclusion of different institutions relevant to aquatic food production in state, market, and civil society. An inter-institutional system is typically stable because institutions tend to be durable or slow to change. However, institutions are also changeable and can thus enable the generation of new and desirable sustainability outcomes (Mahoney and Thelen 2010; Micelotta et al. 2017).

Institutional change is a “phenomenon or a process of change in which institutions undergo a difference in form or quality…over time” (Van de Ven and Hargrave 2004, p. 261, cited in Micelotta et al. 2017). There are various ways in which institutional changes have been classified. Changes may depend on whether they occur in slow-moving (e.g. norms) or fast-moving (e.g. formal regulations) institutions (Roland 2004). Institutional changes have also been classified as designed (deliberate) or evolutionary (absence of a central mechanism coordinating the shift) (Kingston and Caballero 2009), and as developmental (narrow changes) or transformative (discarding of old institutions and replacement with new ones) (Micelotta et al. 2017). Understanding not only how institutions function but also how they change is important for leveraging institutions for sustainability in aquatic food production and other similar sectors. This may require strengthening existing institutions, modifying some, or putting in place new institutions (Galappaththi and Berkes 2014).

To provide a background, the Philippine Fisheries Code of 1998 is the country’s national institutional framework for developing, managing, and conserving fisheries and aquatic resources. This national law and its attendant regulations are primarily implemented by the country’s Bureau of Fisheries and Aquatic Resources (BFAR) in collaboration with other national departments. This policy formally shapes governance at the local level through municipal ordinances (Seki 2009). The existence and content of municipal ordinances vary by context. Formal and new institutions in the aquatic food sector such as certifications exist but are adopted in uneven distribution, and most fisheries regulations focus on capture fisheries. Where aquaculture regulations exist, many are unenforced owing to inconsistencies and lack of capacities to monitor and implement (Guerrero and Fernandez 2018). Moreover, many aspects of aquatic food production and marketing operate outside formal regulations and are under informal arrangements. The inter-institutional system which we adopt as the framework for this research is suitable to the Philippine setting because its systemic approach can cover different spheres of society and include different types of institutions.

## Methods

### General description of the study area

**Fig. 1:**
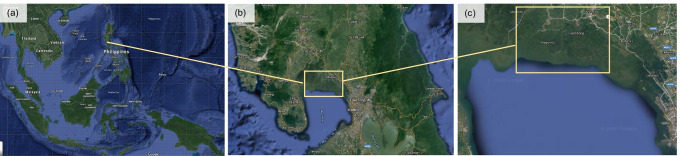
Map of the study area. **a** Southeast Asia showing the Philippines. **b**) The location of the province of Bulacan in Central Luzon. **c** The general location of the case study including the adjacent municipalities of Hagonoy, Paombong, and Malolos along the coast of Manila Bay (source: Google Maps)

The case study is located in the province of Bulacan, within the region of Central Luzon, Philippines. It includes the adjacent municipalities of Paombong, Hagonoy, and Malolos (Fig. 1, Table 1). The three municipalities were selected due to the importance of aquatic food production to the local economy, livelihoods, and food and nutrition security. Moreover, different but connected aquaculture activities are undertaken in the municipalities. Paombong is an important centre for fingerlings production and small-scale aquaculture. Most large-scale aquaculture operations are concentrated in Hagonoy. Both Hagonoy and Malolos are important hubs for fish marketing (Bureau of Fisheries and Aquatic Resources Central Luzon 2017).

**Table 1:** Socioeconomic background of the study area

Characteristics	Paombong	Hagonoy	Malolos
Population	53,510	126,329	223,069
No. of households	8,266	22,174	36,663
Land area (ha)	4,634	10,310	6,725
Area used for brackish water aquaculture (ha)	2,245.6	4,677.5	2,002.9
No. of fishers	579	2,492	830
No. of brackish water fish farmers	286	552	180
Aquatic species farmed	Prawn, milkfish, mudcrabs	Milkfish, prawn, tilapia	Milkfish, prawn, tilapia
Annual brackish water aquaculture production (MT)	259 (prawn) 3,347.6 (milkfish) 13.8 (mudcrab)	14,696 (milkfish) 237 (tilapia) (no data for prawn)	4,005.7 (milkfish) (no data for prawn and tilapia)
Annual production for farmed oyster (MT)	980	1,400	3,750
Average annual catch per fisher	650 kg		
Primary fishing ground	Manila Bay		
Per capita annual fish consumption	27 kg		

Note: Information was taken from the publicly accessible Bulacan Provincial Fisheries Profile for 2017 prepared by the BFAR Central Luzon Office

The coastal area is bounded by Manila Bay. Its resources include municipal waters (i.e. an area 15 km from the shoreline designated for municipal fishing), inland waters, and estuarine ecosystems which are all vital for capturing fisheries of various forms, fingerlings production, brackish water aquaculture in fish ponds, oyster farming, fish cages, and fish pens. Diverse fish species and crustaceans are landed from capture fisheries. The main aquaculture commodities are milkfish (*Chanos chanos*) and tiger prawn species (*Penaeus monodon*). Various tilapia species, mudcrabs (*Scylla serrata*), and vannamei shrimps (*Litopenaeus vannamei*) are also produced. Other livelihood options outside of aquatic food production and diverse commercial activities exist in the municipality centres.

### Data collection and analysis

The choice to adopt a case study approach was based on its suitability to place-based, in-depth, and contextualised investigation of institutions and change processes (Yin 2009; Starman 2013). The unit of investigation and analysis is the coastal social-ecological system consisting of Paombong, Hagonoy, and Malolos. The fieldwork was conducted from November 2019 to early March 2020. Methods for data collection were qualitative and included in-depth interviews, participant observation, and thematic analysis of institutional documents. Interviews started with questions about the interviewee’s demographic profiles, experience in aquatic food production, and about the state of and changes in capture fisheries, aquaculture, and the coastal environment. Interviews then focused on various institutions, and how these changed. Participant observation focused on fishing and aquaculture activities, fish market operations, organisational meetings, and the daily activities of fishers and fish farmers in their communities. Analysis of institutional documents included the Philippine Fisheries Code and the municipal fisheries ordinances of the three municipalities. All methods were used to address the research objectives to enable triangulation. Triangulation means that qualitative content from one data source (e.g. interviews) is compared with other sources (e.g. participant observation, institutional documents) for consistency. This facilitates gaining richer and fuller data and is a way to confirm findings (Wilson 2014).

The selection of interviewees was purposive and combined a snowball and opportunistic approach (Campbell et al. 2020). This means that early interviews provided information about, and connections to other relevant actors who were then subsequently interviewed. A total of 67 interviews were conducted, with a few individuals interviewed more than once for follow-up discussions (Table 2). The selection of interviewees was based on peoples’ active involvement in either capture fisheries or aquaculture and their knowledge concerning conditions and changes in aquatic food production and the broader coastal social-ecological system. The experience of fishers and fish farmers ranged from 3 to over 50 years.

**Table 2:** Sociodemographic characteristics of interviewees

Groups represented	No. of individuals interviewed (by gender)	Age range	Range of educational attainment
State actors	18 (10 M, 8 F)	27–58	High school graduate to master’s degree
Market actors	12 (4 M, 8 F)	42–63	Grade 1 elementary to high school graduate
Fishers	10 (10 M)	39–59	Grade 2 elementary to unfinished college degree
Fish farmers	21 (13 M, 8 F)	38–70	Grade 6 elementary to college graduate
Other fishworkers*	4 (4 M)	45–58	Elementary graduate to high school graduate
NGO and research center	2 (1 M, 1 F)	No data	University degree to doctorate degree

Note: Other fish workers included those who processed fish to produce dried fish and those who provided transport services for fisheries and aquaculture goods

Transcripts from interviews and field notes from participant observations and analysis of policy and regulatory documents were brought together and subjected to qualitative thematic data analysis. The process involved uploading qualitative data into the software MAXQDA Plus 12 (Woolf and Silver 2017) and implementing an iterative coding approach (e.g. Lawless et al. 2019). The coding of qualitative data is a sense-making process in which chunks of text are broken down into categories (Bryman 2012). Here, textual data was first categorised using a preliminary coding tree which included broad categories that were based on the general questions asked during the interviews. The main nodes in the preliminary coding tree included environmental and social changes. The nodes were then expanded by adding sub-nodes or new nodes as more specific themes emerged from the qualitative data. For instance, under the node social change, the sub-node institutions further branched into other sub-nodes such as local regulations, market arrangements, and norms. The early stage of coding revealed that multiple institutions in different spheres of society were important for aquatic food production. Therefore, the second round in the iterative coding applied the inter-institutional system framework and coded institutions according to the three societal spheres of state, market, and civil society (sensu Kooiman and Bavinck 2013) (Fig. S1). The focus was mainly on how institutions in these spheres operate, whether and how they have changed, and due to which drivers.

A limitation of the approach should now be noted. The lack of existing studies on the topic and in the case study meant that a general and open approach to the investigation was needed in order to capture multiple institutions and changes. This required balancing the breadth and depth of investigation (Edwards et al. 2020). Notwithstanding the value of this approach, a more in-depth examination of each of the institutional changes identified is needed to yield insights concerning the role of broader factors beyond the scale of the case study.

## Findings

### The emergence of aquaculture

The case study is a multifunctional social-ecological system that presently supports both capture fisheries and aquaculture. In the past, the population in the study area relied primarily on fishing diverse aquatic organisms in creeks, rivers, estuaries, and in Manila Bay. Rice farming was then common. The spread of aquaculture accelerated in the 1990s amongst households whose rice farms were increasingly affected by environmental change, particularly saline water intrusion (Fig. S2). The conversion from rice farms to fish ponds was initially seasonal. The profitability of aquaculture and increasing salinity levels which made some areas untenable for rice production led to the permanent conversion of many rice farms to fish ponds. Prawn, an exported and high-value commodity, was the main produce. Rearing was done in the traditional manner of using household food scraps and moss as feed. Within community networks, informal sharing of information about aquaculture techniques was an important driver of widespread aquaculture adoption.

In a part of Hagonoy, we heard that they had shifted to fish ponds. They did it first because they were affected by salty water. Those areas were previously rice farms… My sibling from that area recommended that we try to produce prawn. Water had become salty anyway and we were not harvesting rice. (small-scale fish farmer, Paombong)

This shift was enabled first by access to land, and later through lease arrangements. The spread of aquaculture and its subsequent establishment as a dominant aquatic food production sector emerged partly as an adaptive response to environmental change.

Following its widespread adoption, aquaculture production intensified, especially in large aquaculture farms. This was driven by increased demand for and profitability of farmed aquatic food. It was also enabled by access to land either through acquisition or lease, the industrialisation and commercialisation of feed production within the province of Bulacan, increased access to more affordable fry through importation from Indonesia, development of local nurseries for fingerlings production, financing modes, and diffusion of knowledge and technology from both public and private channels.

Aquaculture production in the case study is diverse including smallholder non-intensive and semi-intensive brackish water fish ponds,^3^ large-scale intensive brackish water fish ponds, small fish cages, and more recently, intensive large fish pens towards Manila Bay. Many households continue to depend on capture fisheries for their livelihoods and food, but fish production is increasingly coming from aquaculture.

Water pollution is an environmental problem that undermines the sustainability of both capture fisheries and aquaculture. This is caused by the lack of effective regulation for aquaculture activities resulting in the indiscriminate use of industrial feeds and the disposal of untreated water from fish ponds.

Large proprietors indiscriminately used feeds which damaged not only our fish ponds here but also affected those who are involved in commercial and municipal fishing in the sea… The use is excessive… The problem with this is, when the water from those fish ponds are released to the rivers, naturally occurring species here are adversely affected. (small-scale fish farmer, Paombong)

Water pollution is further exacerbated by neighbouring industrial and domestic sources (Fig. S3). Together, these have led to increased frequency of fish kills in ponds and localised disappearance of aquatic organisms that are valuable for food.

### Institutions, institutional changes, and drivers of change

**Fig. 2:**
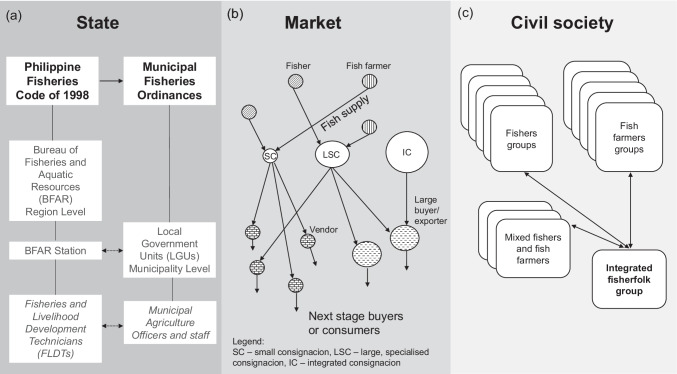
Selected institutions in the spheres of state, market, and civil society which are relevant to aquatic food production. **a** The Philippine Fisheries Code of 1998 (national law) and municipal fisheries ordinances (local rules) are formal state institutions. The Bureau of Fisheries and Aquatic Resources (BFAR) and Local Government Units (LGUs) are the state organisations that are primarily responsible for implementing these regulations. **b** The consignacion is a dominant market arrangement for consolidating fish and other aquatic food from fish farmers and fishers and for selling fish and other aquatic food to vendors and traders as a consignment. Types of consignacions included small consignacions (SC), large, specialised consignacions (LSC), and integrated consignacions (IC) which cater to different types of buyers. **c** The formation of formal and registered fishers, fish farmers’ or mixed (of both) associations have become common forms of local organising. An integrated group was recently formed from different fisherfolk groups to address challenges faced by smallholders especially those related to markets

Following the conceptual framework of the inter-institutional system, the following findings are structured along the sub-sections of state, market, and civil society. In each sub-section, we address the research objectives. Thus, we describe institutions that underpin aquatic food production in the case study, describe whether and how institutions in each of the three spheres changed, and explain key factors that influenced the change processes (Fig. 2, Table 3).

**Table 3:** Summary of institutions, functions, and key changes

Institutional spheres	Institutions and key functions	Description	Changes in the institutional sphere
***State***	Philippine Fisheries Code of 1998 that tasked the Bureau of Fisheries and Aquatic Resources (BFAR) with the management, development, and conservation of Philippine fisheries and aquaculture. In the study area, this translated to: (1) Organisational discourse focused on supporting the poorest in fisheries and aquaculture; and (2) Interventions at the municipal level focused on livelihoods assistance and programs for capacity building and network-building amongst fishers and fish farmers	Multi-scalar structure situated at national and regional levels	Move towards local embedding and multi-actor coordination through the establishment of a provincial station and FLDTs New approach to a more focused targeting of assistance for small scale fisheries and aquaculture actors HACCP and GAP certifications for large scale aquaculture Move towards formalisation of fisheries and aquaculture through Fish R and Boat R
	Municipal Fisheries Ordinances implemented by Local Government Units which are oriented towards regulatory enforcement	Devolved jurisdiction over inland and municipal waters, leadership in fisheries and aquaculture is co-terminus with terms of elected political officials	Implementation of auxiliary invoices and local transport permits
***Market***	Consignacions: (1) Structure market exchanges for fish and other aquatic food; (2) Node connecting fish farmers and fishers to buyers (3) Risk-redistribution mechanism (4) Alternative credit provider	Businesses are registered with a local government unit Embedded in local social relationships Linked with external and wider market networks through traders Operates through suki relationships Asymmetric power relations entrenched through utang as a social practice Minimal state intervention (i.e. handling of complaints, infrastructure support, conflict resolution)	Bidding to pre-arranged purchases Bidding to non-bidding mechanisms or manipulations Emergence of large, specialised consignacions Emergence of integrated consignacions (production and marketing) Emergence of alternative market routes (e.g. purchase at sea)
***Civil society***	Associations of fishers/farmers/mix of both Integrated fishers’ and fish farmers’ association	Formal, registered associations	Emergence of fishers’ and fish farmers’ associations as the norm Emergence of integration of different associations

#### State—moving towards local embeddedness and multi-actor coordination

As earlier mentioned, state governance of aquatic food production in the Philippines involves the national government and local government units (LGUs). The distinctive roles of the national and local governments are reflected in two-pronged institutions for aquatic food production; one oriented towards sectoral development and the other towards formal regulation (Fig. 2a). The role of BFAR, a national department, is mainly development-oriented (e.g. livelihood assistance, capacity building), while that of the municipal LGUs is oriented towards local regulatory enforcement (e.g. issuance of permits and collection of fees, monitoring, penalties). In practice, there is significant overlap in the activities of these two actors and their interactions within governance structures contributed to the change dynamics described below.

The Philippine Fisheries Code (Republic Act 8550) of 1998 established and tasked BFAR at the national level with the management, development, and conservation of Philippine fisheries, including aquaculture. To align with the Local Government Code passed earlier in 1991 (Republic Act 7160), the fisheries code devolved the management and regulation of municipal waters and inland waters used for municipal fishing and aquaculture to LGUs headed by elected mayors. The LGUs in the study area adopted and contextualised the national fisheries code through the creation of their respective municipal fisheries ordinances at different junctures (i.e. 2000 for Malolos, 2009 for Hagonoy, and ongoing for Paombong). The process of formulating local fisheries ordinances was intended to ensure that nationally legislated rules were suited to the context. For instance, that penalties were not excessively high. Small-scale fish farmers viewed the time lag in contextualisation to be indicative of the extent to which the formal development of fisheries and aquaculture regulations is subjectively dependent on political will.

Thematic analysis of approved municipal fisheries ordinances in both Hagonoy and Malolos revealed a path-dependent and disproportionate focus on capture fisheries including rules around fishing gears, specifications and limitations of fishing activities in various areas, penalties for violations, and permit and fee requirements. Except for a prohibition against disposing of toxic chemicals (e.g. sodium cyanide) into water bodies which also applied to capture fisheries, there was a lack of regulation for aquaculture production, both *de jure* and de facto. Small-scale fishers and fish farmers viewed factors such as LGU officials’ personal involvement in intensive aquaculture production, close ties with large-scale aquaculture producers, and desire to gain votes for election as some of the reasons maintaining the gap in aquaculture regulation. In addition, the shared narrative and perception around aquaculture as ‘private property’ which was observed among fish farmers, fishers, and government representatives reinforced the mode of minimal to no state regulation.

One would think it’s just a body of water. But you find out later that the area is titled private property. They pay property tax. That becomes an issue. (government employee, Hagonoy)

BFAR has a national scope and carries the mandate of promoting food security contained in the country’s fisheries code. Discourse in the organisation is focused on supporting the poorest in the fisheries and aquaculture sectors. This discourse is translated into productivity-focused projects at the municipality level for both smallholder fish farmers and fishers. BFAR moved towards a more targeted selection of so-called project beneficiaries with the aim of improving its effectiveness in channelling support to the poor. For instance, it supports smallholder aquaculture by distributing fingerlings, conducting capacity-building activities (e.g. short course on aquaculture green water technology), improving access to low-interest financing, and providing support to fish farmers and fishers to establish connections with new markets. Institutions targeted for the development of large-scale aquaculture are different. These included certification schemes (e.g. Hazard Analysis Critical Control Point or HACCP, and Good Aquaculture Practices or GAP) aimed at developing producers’ competitiveness in large urban markets and export.

Our findings revealed that while local regulatory institutions were less responsive to the emergence of aquaculture as an important aquatic food production sector, development-oriented institutions were relatively more responsive. The move towards better targeting of beneficiaries was driven by the need to respond to what BFAR perceived as shortcomings in the delivery of effective livelihood support for smallholder fish farmers and fishers. A BFAR official explained that previously, its mode of operation at the municipality level which depended on turning its livelihood assistance over to local politicians was riddled with problems. These included the diversion of livelihood assistance to political allies and relatives in order to strengthen patron-client relationships and gain voters’ support for subsequent elections. This was one of the plural ways in which formal institutions in aquatic food production were entangled with local political dynamics. To address this and related problems, BFAR implemented a strategy for local embedding and closer coordination with LGUs and other actors.

From the mid-2010s, institutional change within BFAR involved the creation of a network of Fisheries and Livelihood Development Technicians (FLDTs). FLDTs are BFAR staff who liaise with local government units and with organised groups of fish farmers and fishers in communities. Their functions helped influence BFAR’s responses to fish farmers’ and fishers’ needs, monitor impacts of livelihood assistance projects, and inform collaborations with LGUs. The level of local embeddedness enabled by this new institution contributed to the emergence of further collaborations and the reinvigoration of rules which had been stipulated in the national fisheries code but until recently remained under-implemented at the municipality level. These included Fish R and Boat R which are registry programs requiring all fishers, fish farmers, and their boats to be formally registered. These programs have since been co-implemented by BFAR and the LGUs. Auxiliary invoices and local transport permits are also among the newly co-implemented rules. These were designed to track fishery and aquaculture goods transported out of Hagonoy and Malolos where the largest fish markets are located. Because most goods transported out of the study area are from aquaculture, these rules provided an alternative means for BFAR and LGUs to estimate aquaculture production in the municipalities.^4^

#### Market—the centrality of consignacions

In the market sphere, the term *consignacion* comes from the word consignment which is generally understood as a market arrangement in which goods are taken and paid at a later point. In the study context, the term consignacion refers to registered businesses that continually purchase aquatic food from fish farmers and fishers throughout the day and sell these through an auction on a rolling basis. More than a reference to the central market actors, the term consignacion signifies the primary structure and dominant institution shaping market exchanges. The consignacion is strongly embedded in long-standing and durable social relationships. Consignacions in the study area are believed to have started long before the 1970s as a person-to-person arrangement intended to relieve fishers of the time and effort required to sell their catch and as a way to ascertain that the catch is sold. Fish and other aquatic organisms used to be sold in heaps or basins, before they were sold in kilograms. The niche has since evolved as a central node in a network of fish producers and buyers who compete for the supply of aquatic food (Fig. 2b). Consignacions preceded the expansion and intensification of aquaculture and initially existed for capture fisheries. Fish and other aquatic food from aquaculture were assimilated into the pre-existing arrangement in fish markets.

Social relationships between smallholder producers and consignacion business owners typically involved features of patron-client relationships. This applied particularly to poor fishers who have limited alternatives for accessing finance. These social relationships served as sites for enactments of informal and cultural norms such as *utang* (borrowing and lending money informally) and *suki* (regularity in market exchanges which eventually fosters familiarity and trust). Thus, depending on the social and economic position and needs of the smallholder producer, relationships with consignacions were characterised by asymmetric power relations predicated on economic leverage. Utang in which money is lent to fishers and fish farmers as a form of livelihood assistance (e.g. for the repair of boats, purchase of feeds) or to help meet household needs, functioned as a means through which consignacions controlled fishers and fish farmers by locking smallholder producers into informal arrangements in which they can only sell to the consignacions they were indebted to until the debt was paid in full. This had the effect of closing any opportunity to sell to others for a better price. With many fish producers particularly capture fishers struggling to make ends meet while having limited access to financial services, utang particularly from consignacions became perennial. This had a substantive impact on the livelihoods and income of smallholder producers. As a de facto rule, fishers and fish farmers who sold aquatic food to consignacions without standing debt were formerly paid 5–6% less than the actual price of goods. This had now increased to about 8–9%. Under this arrangement, fishers and fish farmers were locked into receiving only a fraction of the full value of their goods. The word locally used to denote this is “percentage”, that is, the value appropriated to the consignacions. Fish producers with debts typically lost more. This arrangement was underpinned by people’s collective and tacit view that the “percentage” is a business owners’ right to profit.

Fish and other aquatic products sold by consignacions to traders or vendors were paid by the latter when the goods had been sold to the next level of the supply chain (e.g. to processors, to consumers). The time lag for payment varied between 3 days or more, depending on subjective and negotiated factors. The agreement was conditional, and pay-later arrangements applied only for suki who are trusted because of familiarity gained through repeated transactions. New buyers who were not yet suki were required to pay in cash immediately.

Consignacions provided a risk re-distribution service which relieved fish producers of the responsibility to market their produce. They provided fishers with the assurance that their fish will be sold and enabled buyers down the supply chain to access aquatic food without the need for outright payment. Under this institutional market arrangement, however, small-scale fishers and fish farmers were locked into receiving lower benefits from food production, relative to other market actors, while also elevating the fish price paid by consumers.

Buyers of fish such as local vendors and regional traders competed in fish markets through on-the-spot auctions where bids were traditionally indicated through whispers, and where the highest bidder bought the fish. However, such a process had also been manipulated by consignacions to favour certain sukis, to strengthen reciprocal market ties, or to artificially elevate the price of aquatic food for profit. A few adjustments had been made to the bidding process (e.g. from whispers to bids written down) but the bidding dynamics remained largely the same.

In the market sphere, two key institutional changes developed from the intensification of aquaculture. These were the development of large-scale and specialised consignacions, and the establishment of consignacions that were integrated with aquaculture production. The first differed from the small consignacions because they have specialisation in high-value aquaculture goods such as prawn and milkfish. Utang was also practiced between large consignacions and fish farmers who were considered to have the capacity to repay. In specialised consignacions, aquatic foods from aquaculture were typically graded and priced according to size. These consignacions tended to engage in bigger-volume and higher-value transactions, including with exporters. Transactions still involved auctions but variations were increasingly adopted to elevate the auction bids, fish prices, and profit. Mobile phones have increasingly enabled distant product orders and price agreements particularly with large buyers, outside of established auction practices. The second institutional change involved the biggest aquaculture producers in the area who established their own consignacions in order to link directly with large buyers and avoid losses from standard consignacion “percentage.”

On the other hand, smallholder fish farmers and fishers have been less able to establish alternative and advantageous market arrangements. A subversive practice to avoid consignacions involved fish processors setting out to sea to buy directly from fishers’ boats. However, such practices remained marginal market practices.

#### Civil society—organising and collective action

The formation of fisherfolk associations (Fig. 2c) was stipulated in the national fisheries code since 1998. Fisherfolk associations were led by an elected group of officials and operated under a set of by-laws. These by-laws were typically patterned after other formally registered groups and were adopted to suit the purposes of new groups. Fisherfolk associations functioned independently from one another but were loosely networked at the municipality and provincial levels through the Municipal Fisheries and Aquatic Resources Management Councils (MFARMCs) and the Bulacan Fisheries Consultative Council (BFCC), respectively. These councils were intended to provide space for selected representatives of fisherfolk organisations to participate in governance-related discussions. In terms of its operations, the associations functioned as nodes for formal and informal collaborative activities and as a basis for recognition and source of legitimacy which enabled fisherfolk to access state services.

The establishment of new associations was viewed by fish farmers and fishers as being linked with the state’s initiative to improve the targeting of project beneficiaries which required fish farmers and fishers to formally organise. For instance, the formulation and submission of requests for livelihood support were partly contingent on membership in a formally recognised association. This requirement provided the impetus for associations to develop as an established social practice for local organising and collective action. The associations included new groups of fish farmers who either organised based on their geographic proximity (i.e. neighbours), or their shared experience in state-organised aquaculture training. These associations played an important role in the diffusion of new aquaculture-related information, technology (e.g. green water technology), and practices through discussions and information campaigns within their communities. For example, several fish farmers who were interviewed reported a change in practice from the use of the highly toxic and prohibited sodium cyanide to the government-prescribed teaseed powder during pond preparation. Moreover, these associations served as hubs for the sharing of information about the sourcing of fingerlings, organic feed, and input and output market prices. Telecommunications and social media were important drivers of information sharing not only between members of organised groups, but also with state actors. The majority of interviewees viewed the formation of organisations as having contributed to amplifying their voices and enabling them to gain recognition and support from state actors. This was viewed as a positive change relative to past conditions. Organisations maintained their cohesion through regular monthly meetings which commonly became venues for shared meals and social interactions.

In 2018, nine organisations consisting of a mix of smallholder fishers and fish farmers in the study area formed a larger and integrated fisherfolk association. The integrated association worked with state actors to initiate the operation of a new Community Fish Landing Center (CFLC). This was enabled by the association’s establishment of a cooperative that allowed fish farmers and fishers to pool their resources including capital and labour with the aim of gradually building capacity in marketing and reducing dependence on consignacions. While the CFLC faced operational problems, the group started to establish new market linkages and to attempt to expand their market activities in other urban areas including Metro Manila.

## Discussion

The findings identified and described institutions in the spheres of state, market, and civil society which influenced aquatic food production, whether and how they changed, and key factors that influenced the changes. In the sphere of the state, key institutions include formal government structures (i.e. BFAR, LGUs) and associated fisheries regulations at the national and local scales (e.g. Fisheries Code of the Philippines, municipal fisheries ordinances). In the sphere of market, the most important institution is the selling and buying arrangement centred around consignacions or middlemen. In civil society, the key institution involves fishers’ and fish farmers’ organisation as the norm of local organisation and collective action. Similar to findings of other studies around the governance of the coastal realm (e.g. Van Assche et al. 2020), these institutions underwent changes that were developmental in scope, characterised by modifications and extensions of already existing institutions and not by displacement (Micelotta et al. 2017). Changes in state institutions were driven by the need to address challenges in government processes, while the contextualisation of national fisheries law into local regulations was strongly shaped by local political interests. New institutions for trading aquaculture goods in the form of specialised consignacions and consignacions integrated with fish farms were directly driven by aquaculture growth. The new norm around local organisation was driven by formal government institutions, the need of small-scale aquatic food producers to gain recognition, legitimacy, and access to government service, and by social connectedness in communities.

The findings demonstrate how social and ecological sustainability outcomes in the context of aquatic food are embedded in an inter-institutional system consisting of multiple and dynamic institutions (De La Torre-Castro and Linström 2010; Schlüter et al. 2020; Trung Thanh et al. 2021). The account we presented is in line with what Cleaver and Whaley (2018, p. 6) described concerning how “the evolution of governance arrangements over time, the coexistence of multiple institutions at different scales, differences of understandings and inequalities of power between stakeholders, all interact to produce a complex governance scenario.” Thus, the challenge of realising multi-dimensional sustainability outcomes (e.g. food and nutrition security, equity, environmental health) in dynamic aquatic food systems needs to move beyond the focus on technology development, transfer of best practices, or even redesigning institutions for sustainability. Fundamentally, sustainable governance of aquatic food systems requires an empirical and context-specific understanding of the inter-institutional system in which the food system is embedded (sensu Jentoft and Chuenpagdee 2009; Jentoft 2018). This can help determine where institutions are ineffective, missing, or generating undesirable outcomes (e.g. pollution, inequities) as a basis for food system actors to act on. Being embedded in an inter-institutional system, an institution does not generate outcomes in isolation. In this case, formal state regulations influenced the emergence of local organisation in civil society, which in turn may help spur alternative market arrangements. Thus, it is necessary to attend to the way that configurations of and linkages between multiple types of institutions in different spheres of society shape sustainability challenges and their solutions (e.g. McGinnis 2011; Galappaththi and Berkes 2014; Cleaver and Whaley 2018).

It should be noted that institutions do not necessarily have a one-to-one correspondence with outcomes, and that institutions designed for a specific purpose have evidently been used for other purposes in processes of bricolage (Cleaver and De Koning 2015). An important governance implication is that multi-dimensional sustainability is not likely to be achieved by any single designed institution (e.g. aquatic food certification, regulations) but through engagement with the relevant system of salient institutions in any given place. In the same vein, Bush and colleagues (2019b) therefore advocate for the governance of aquaculture innovation that takes into account social, economic, and political contexts.

Because multiple institutions that underpin aquatic food production exist in different forms which evolve and change through different mechanisms and in response to different drivers, the extent to which sustainability can be achieved by directly leveraging institutional change (sensu Meadows 1999) is significant but has limitations (Cleaver and De Koning 2015). Sustainability in aquatic food production and similar other sectors is likely to depend on contextually suitable combinations of effectively designed institutions (Jentoft 2018), and the presence of enabling conditions for desirable institutions to evolve out of emergent needs or opportunities (Jentoft 2007). In relation to this, Meadows (2008) discussed the advantage of integrating learning into how institutions operate and develop by allowing institutions to adjust under changing conditions. Because evolutionary institutional change is not directly amenable to being designed, further research is needed to investigate the extent to which evolutionary institutional changes can be supported to integrate learning cycles in the long term. In the case of aquaculture growth, what supportive factors will need to be put in place so that evolutionary institutional changes can better contribute towards the achievement of normative sustainability goals?

Our findings showed that social relationships shape change processes. Cohesive social relationships between fish farmers and fishers at the community level, and between community and state actors contribute to enabling conditions for beneficial institutions and institutional change (Galappaththi and Berkes 2014). Such grassroots and community-driven processes are valuable because they promote inclusivity and community participation, allowing local actors to actively take part in shaping social arrangements (see Jentoft 2020 for the importance of coastal communities). In turn, formal rules can contribute to fostering such social connections and cohesion.

Furthermore, institutional change does not occur in a vacuum but is political (Chang 2007). The gap in aquaculture regulation is tacitly influenced by the distribution of power among actors, aligning or competing interests, and alliances (e.g. Verbrugge 2015). Due to the devolved nature of coastal and marine governance in the Philippines, local political dynamics play a highly significant role in shaping regulations (Seki 2009). The interests of politicians in maintaining votes and the political influence of large-scale intensive aquaculture producers serve to maintain the regulatory gap in aquaculture despite the increasing degradation of the natural environment in which local livelihoods and industry depend. Institutional change by design therefore needs to engage with power asymmetries (Cleaver and De Koning 2015; Bennett et al. 2018).

## Conclusion

Multiple institutions in different societal spheres contribute to outcomes in aquatic food production (Nayak and Berkes 2014; Partelow et al. 2018). The broad coverage of the inter-institutional system used in this research helps identify different areas of institutional gaps, inadequacies, and ineffectiveness. At the same time, it also reveals areas of strengths that governance actors can build on (e.g. existing collaborations) (e.g. Galappaththi and Berkes 2014). Two key areas stand out as urgently needing action. The first is environmental degradation caused by water pollution. Strict, contextually suited, and effectively enforced regulation of aquaculture practices to curb the excessive use of commercial feed, and to incentivise treatment and proper disposal of polluted water from ponds and fish pens are needed. Institutions for aquatic food production at different scales and particularly at the municipal level need to respond to old and new sustainability challenges brought about by the expansion and intensification of aquaculture (Techera and Hassan 2021). Along this line, it is highly needed to distinguish between polluting activities of large- and small-scale producers and develop suitable standards. The second is inequitable distribution of benefits. Smallholder aquatic food producers receive the least benefit from aquatic food production. Achieving the Sustainable Development Goals of ending poverty and zero hunger and malnutrition necessitates taking proactive steps to address inequity, including through transforming inequitable market institutions (Thilsted 2021; Campbell et al. 2021; Brugere et al. 2021).

Details in the “Findings” section provide insights into some of the factors that need to be considered. First, given the current structure of fisheries and aquaculture governance, municipal fisheries ordinances that are promptly responsive to aquaculture development, and not stalled by political interests, are paramount. However, a change in formal regulation alone may not be sufficient (sensu Abson et al. 2016; Manlosa et al. 2018). As shown in the “Findings” section, the shared mindset around *aquaculture as private property* is capitalised by large-scale producers to prevent any semblance of monitoring, prevent government staff from engaging with large-scale producers, and blunt smallholders’ demand for the regulation of polluting practices. This mindset needs to be changed through collective critical reflection. Second, there is a need for government to be more intentionally engaged in market arrangements and to develop policies that promote equitable arrangements (Brugere et al. 2021). Since consignacions offer a risk-redistribution mechanism and financial assistance that is vital to producers, an immediate overhaul of the arrangement may not be tenable. However, smallholders may be supported to strengthen their market position by organising as a cooperative, exploring new market avenues, and experimenting with alternative market arrangements that will work best for their context (Wegerif 2020; Manlosa et al. 2021). To make this possible, organised groups will need access to financial grants, adequate and low-interest loans, and connections with other market actors beyond the producers’ immediate locality.

## Supplementary Information

Below is the link to the electronic supplementary material.

Supplementary file1 (DOCX 48 kb)

Supplementary file2 (DOCX 859 kb)

Supplementary file3 (DOCX 1879 kb)

## Footnotes

1 In some cases, the term institution is used to refer to an organisation. North (1990) and Jentoft (2004) discuss this issue. Our definition of institution refers to rules, and therefore extends beyond organisations. However, in line with Jentoft (2004), formal organisations such as state departments and offices are structured and operate based on bundles of rules. We therefore also consider organisations as a type of institution, among many other types.

2 We use the term aquatic food to broadly refer to fish and other organisms from capture fisheries and aquaculture used for consumption. Others refer to it as blue food.

3 There was no formal basis for classifying small scale and large scale fishpond aquaculture. However, fish farmers and government staff viewed small scale aquaculture to include those with 1–10 has. of land. Large scale aquaculture varied from tens of hectares to as much as a thousand hectares.

4 Compliance was serendipitously aided in 2019 by the outbreak of African Swine Flu which led to more stringent monitoring of movement of various commodities at road checkpoints, and in 2020 by the COVID-19 pandemic which intensified regulation of movements.
