# Supplementary material for: Institutions and institutional changes: aquatic food production in Central Luzon, Philippines
Source: Reg Environ Change. 2021 Dec 2;21(4):127. doi: 10.1007/s10113-021-01853-4 (PMC8637508; doi:10.1007/s10113-021-01853-4)
Supplement: Supplementary file 1 — Supplementary file1 (DOCX 48 kb) [file 10113_2021_1853_MOESM1_ESM.docx]

**A schematic of the preliminary coding tree**

Various types and change processes

Fisheries-related changes

Aquaculture-related changes

Institutions and changes in institutions

Livelihood shifts

Saline water intrusion

Worsening water pollution

Land use change / farms converted to fish ponds

**Social changes**

**Environmental changes**

**A schematic of the axial coding tree**

**Institutions and change processes in the sphere of state**

**Institutions and change processes in the sphere of market**

**Institutions and change processes in the sphere of civil society**

**Fig. S1** Schematic diagram of coding tree.
