# Supplementary material for: Institutions and institutional changes: aquatic food production in Central Luzon, Philippines
Source: Reg Environ Change. 2021 Dec 2;21(4):127. doi: 10.1007/s10113-021-01853-4 (PMC8637508; doi:10.1007/s10113-021-01853-4)
Supplement: Supplementary file 2 — Supplementary file2 (DOCX 859 kb) [file 10113_2021_1853_MOESM2_ESM.docx]

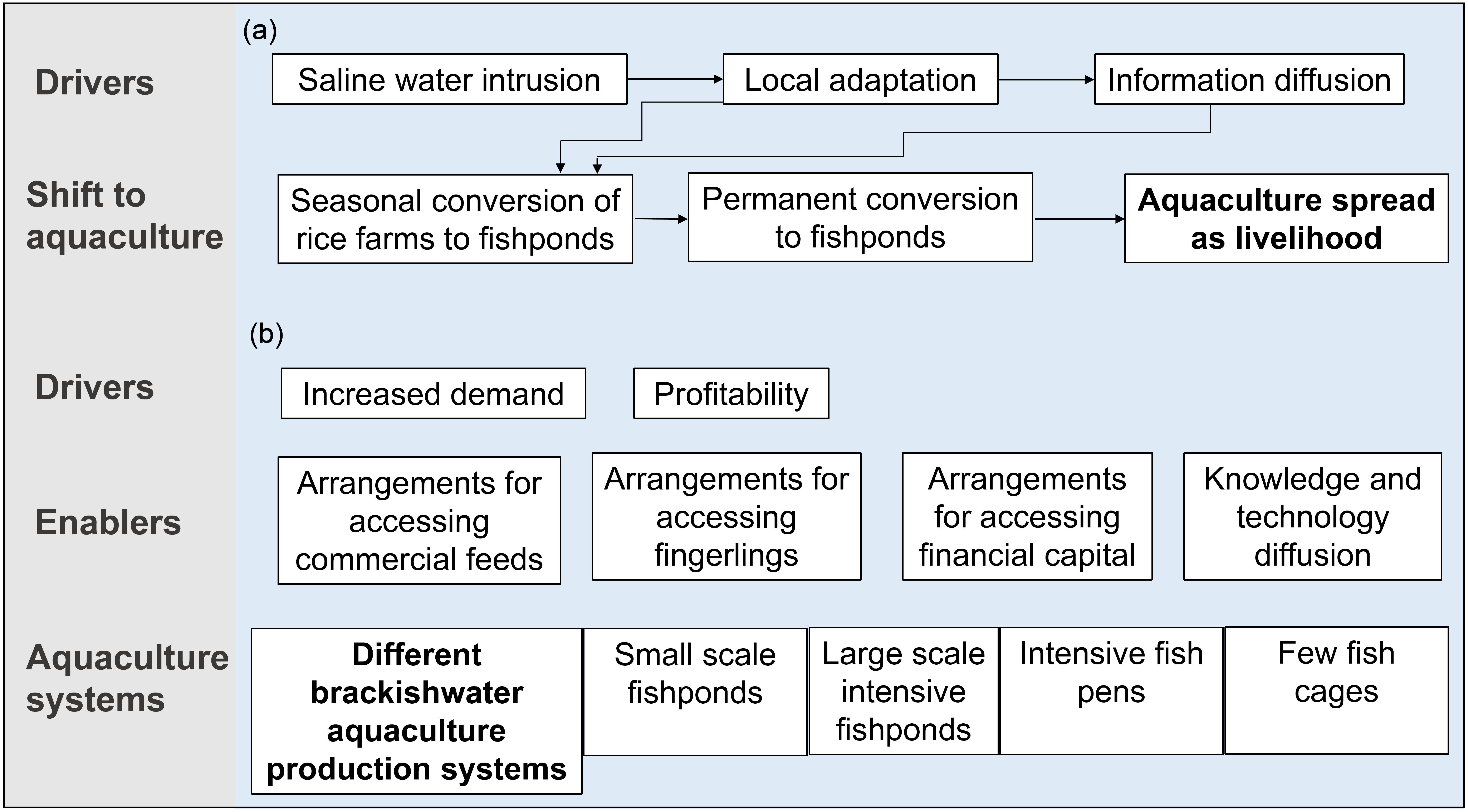


**Fig. S2** (a) Factors influencing shift to aquaculture as a livelihood due to environmental change. (b) Factors contributing to the establishment of different aquaculture systems including intensive aquaculture.
