# Supplementary material for: Institutions and institutional changes: aquatic food production in Central Luzon, Philippines
Source: Reg Environ Change. 2021 Dec 2;21(4):127. doi: 10.1007/s10113-021-01853-4 (PMC8637508; doi:10.1007/s10113-021-01853-4)
Supplement: Supplementary file 3 — Supplementary file3 (DOCX 1879 kb) [file 10113_2021_1853_MOESM3_ESM.docx]

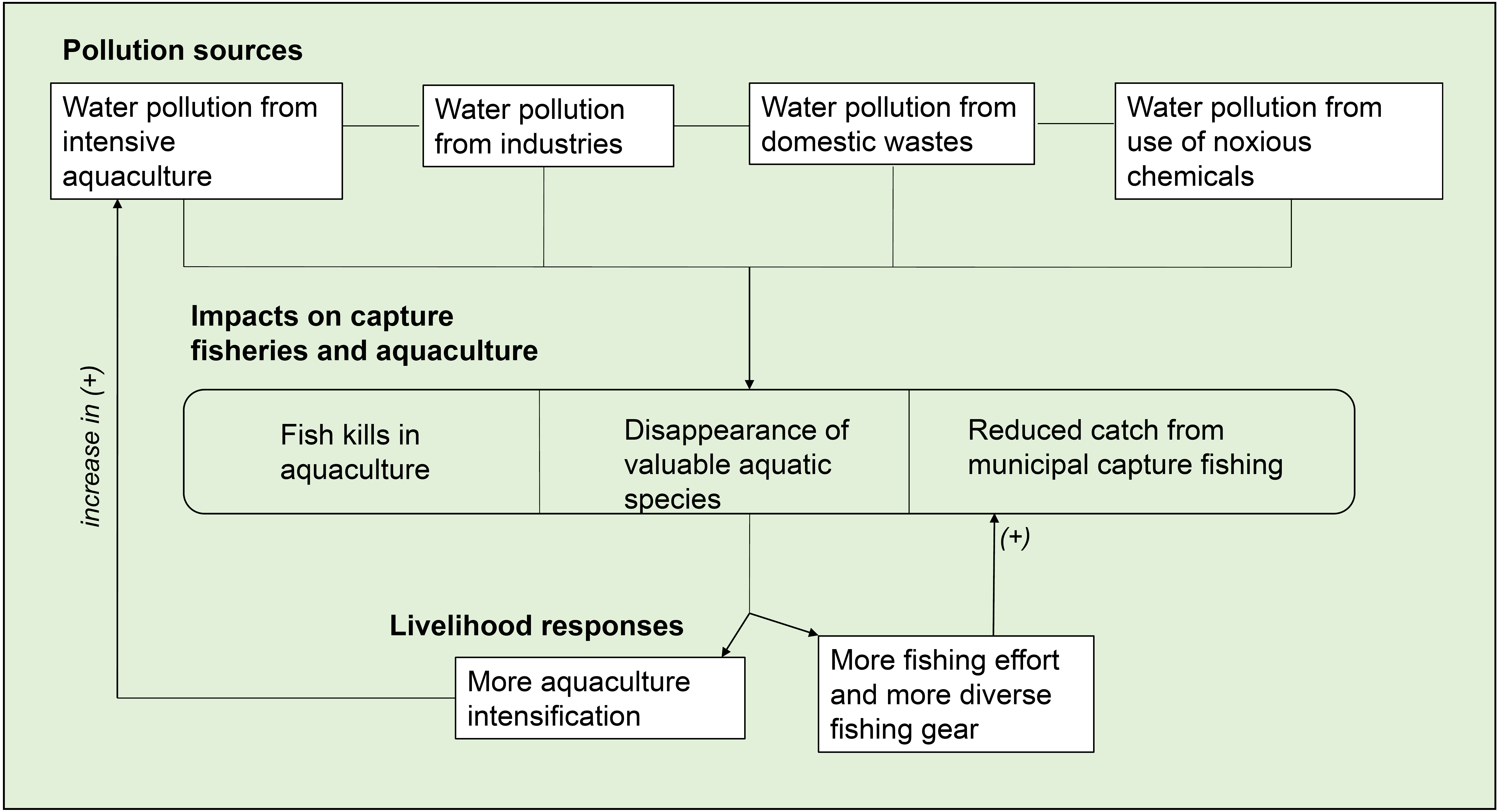


**Fig. S3** Water pollution was the most commonly mentioned environmental problem which negatively affected capture fisheries and aquaculture. The figure shows effects of water pollution and positive feedbacks.


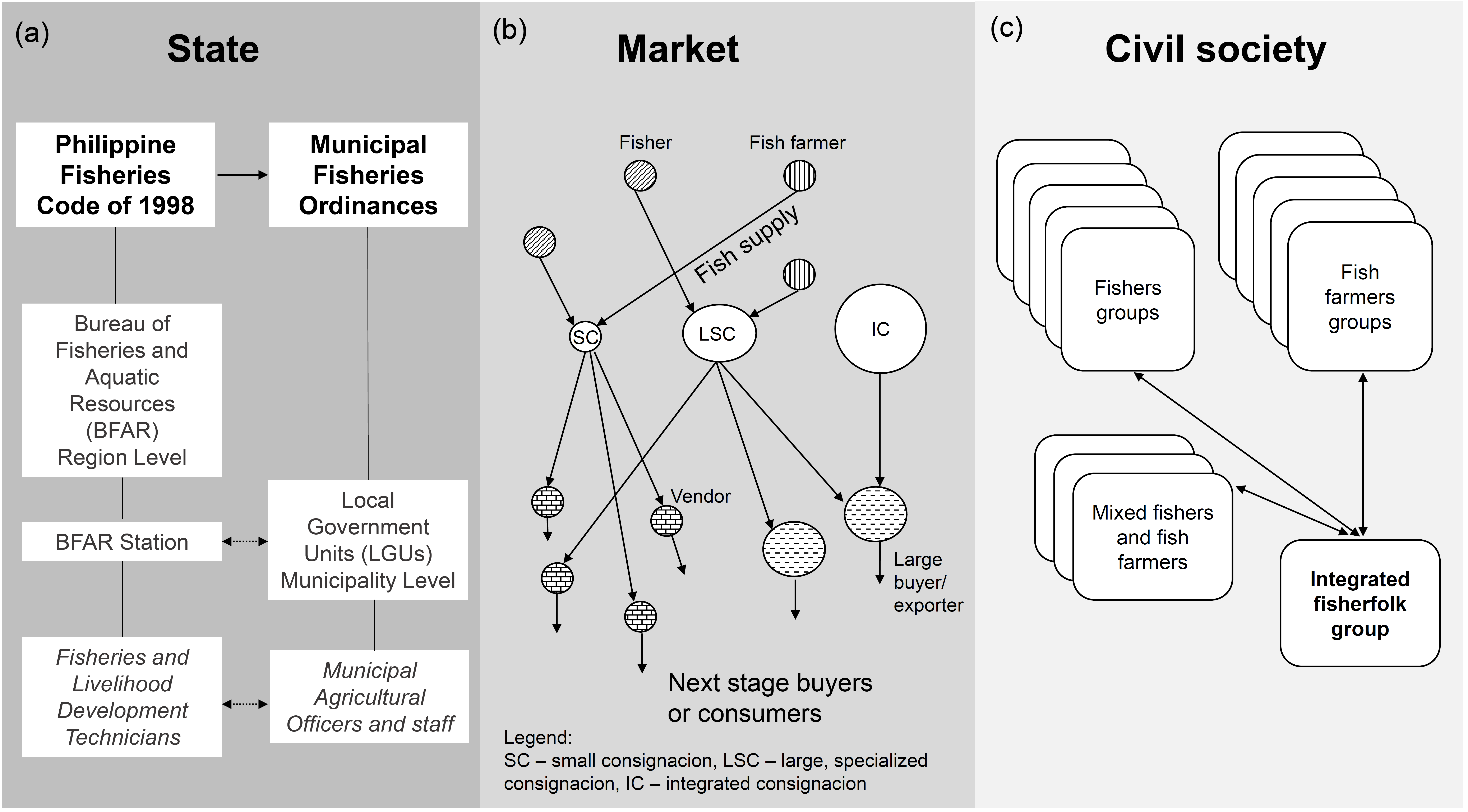


**Fig. 3** Selected institutions in the spheres of state, market, and civil society which are relevant to fisheries and aquaculture. (a) The Philippine Fisheries Code of 1998 (national law) and municipal fisheries ordinances (local rules) are important institutions. The main responsibility for these institutions are with BFAR and LGUs. (b) The consignacion is a dominant market arrangement for consolidating fish and other aquatic food from fishers and fish farmers and selling these to vendors and traders as consignment. Types of consignacions included small consignacions, specialized consignacions, and integrated consignacions which cater to different types of buyers. (c) The formation of formal and registered fishers, fish farmers’ or mixed (of both) associations have become a norm in local organization. An integrated fisherfolk group in Paombong was recently formed from different groups.
